# Global Control and Regional Elimination of Measles, 2000–2011

**Published:** 2013-01-18

**Authors:** Robert T. Perry, Marta Gacic-Dobo, Alya Dabbagh, Peter M. Strebel, Jean-Marie Okwo-Bele, James L. Goodson

**Affiliations:** Dept of Immunization, Vaccines, and Biologicals, World Health Organization, Geneva, Switzerland. Div of Viral Diseases, National Center for Immunization and Respiratory Diseases; Global Immunization Div, Center for Global Health, CDC

Widespread use of measles vaccine since 1980 has led to a substantial decline in global measles morbidity and mortality; measles elimination* has been achieved and sustained in the World Health Organization (WHO) Region of the Americas (AMR) since 2002. In 2010, the World Health Assembly established three milestones for measles eradication to be reached by 2015: 1) increase routine coverage with the first dose of measles-containing vaccine (MCV1) for children aged 1 year to ≥90% nationally and ≥80% in every district or equivalent administrative unit; 2) reduce and maintain annual measles incidence to <5 cases per million; and 3) reduce measles mortality by 95% from the 2000 estimate (1). The Global Vaccine Action Plan (GVAP) includes monitoring progress toward achievement of goals to reduce or eliminate measles in four WHO regions by 2015 and five WHO regions by 2020 (2).† This report updates the previous report (3) and describes progress in global control and regional elimination of measles during 2000–2011. Estimated global MCV1 coverage increased from 72% in 2000 to 84% in 2011, and the number of countries providing a second dose of measles-containing vaccine (MCV2) through routine services increased from 97 (50%) in 2000 to 141 (73%) in 2011. During 2000–2011, annual reported measles incidence decreased 65%, from 146 to 52 cases per 1 million population, and estimated measles deaths decreased 71%, from 542,000 to 158,000. However, during 2010–2011, measles incidence increased, and large outbreaks of measles were reported in multiple countries. To resume progress toward achieving regional measles elimination targets, national governments and partners are urged to ensure that measles elimination efforts receive high priority and adequate resources.

## Immunization Activities

WHO and the United Nations Children’s Fund (UNICEF) use annual data from administrative records and surveys reported by national governments to estimate MCV1 coverage among children aged 1 year.§ Since 2003, countries also have reported the number of districts with ≥80% MCV1 coverage. During 2000–2011, estimated global MCV1 coverage increased from 72% to 84%; for 2011, estimated MCV1 coverage in three WHO regions was ≥90% (Table 1). The number of countries with ≥90% MCV1 coverage increased from 83 (43%) in 2000 to 123 (63%) in 2011. Of countries reporting district-level MCV1 coverage, the proportion reaching ≥80% MCV1 coverage in ≥80% of districts increased from 49% (72 of 148) in 2003 to 56% (87 of 156) in 2011; in 2011, 34% (53 of 156) reported ≥80% MCV1 coverage in all districts. Of the estimated 20.1 million infants who did not receive MCV1 in 2011 through routine immunization services, 11.1 million (55%) were in five countries: India (6.7 million), Nigeria (1.7 million), Ethiopia (1.0 million), Pakistan (0.9 million), and the Democratic Republic of the Congo (DRC) (0.8 million).

During 2000–2011, the number of countries providing a second dose of measles-containing vaccine (MCV2) through routine services increased from 97 (50%) to 141 (73%). Overall, 225 million children received measles vaccination during 39 supplemental immunization activities (SIAs)¶ conducted during 2011. Among those 39 SIAs, 17 (44%) had >95% reported measles vaccine coverage, 12 (31%) included rubella vaccination, 15 (38%) included oral polio vaccination, and 14 (36%) included one or more child health interventions, in addition to vaccinations (Table 2).

## Disease Incidence

During 2000–2011, the number of countries reporting annual measles surveillance data to WHO increased from 169 (88%) to 188 (97%). Effective measles surveillance includes case-based surveillance with laboratory testing to confirm cases. During 2004–2011,** the number of countries using case-based surveillance increased from 120 (62%) to 182 (94%).†† During 2000–2011, the number of countries with access to standardized quality-controlled testing through the WHO Measles and Rubella Laboratory Network increased from 71 (37%) to 191 (98%).§§

During 2000–2011, the number of measles cases reported worldwide each year decreased 58%, from 853,480 to 354,922, and measles incidence decreased 65%, from 146 to 52 cases per million population per year, with declining cases and incidence reported in all WHO regions (Table 1). During 2000–2011, AMR maintained measles incidence at <5 cases per million; in 2011, reported incidence in the Western Pacific Region (WPR) was 12 cases per million, a historic low (Figure). However, since reaching a low of 278,417 reported cases worldwide in 2008, annual reported cases have increased each year. From 2010 to 2011, a decrease in reported measles cases in WPR, from 49,460 to 21,050 cases, was offset by increases in reported cases, from 10,072 to 35,923 in the Eastern Mediterranean Region (EMR), 52,529 to 65,161 in the South-East Asia Region (SEAR), 186,675 to 194,364 in the African Region (AFR), and 30,625 to 37,073 in the European Region (EUR). In addition, the percentage of countries with reported measles incidence <5 cases per million population decreased, from a high of 122 (67%) of 183 reporting countries in 2008 to 104 (55%) of 188 reporting countries in 2011. During 2011, large measles outbreaks were reported by DRC (134,042 cases), India (29,339), Indonesia (21,893), Nigeria (18,843), Somalia (17,298), France (14,949), Zambia (13,324), Chad (8,650), Philippines (6,538), Sudan (5,616), Italy (5,189), Pakistan (4,386), Romania (4,189), Spain (3,802), Uganda (3,312), Ethiopia (3,255), and Afghanistan (3,013).

## Mortality Estimates

Many countries, particularly those with the highest disease burden, lack data on the number of measles deaths; therefore, WHO has developed a model to estimate mortality using reported numbers of cases, measles vaccination coverage through routine vaccination and SIAs, the age distribution of reported cases, and age-specific, country-specific case-fatality ratios (4,5). The addition of 2011 measles vaccination coverage and case data for all countries, and updating of data for the period before 2011 for some countries, led to new mortality estimates for 2000–2011. During 2000–2011, estimated measles deaths decreased 71%, from 542,000 to 158,000; all regions and India had substantial reductions in estimated measles mortality, ranging from 36% to 90% (Table 1).

### Editorial Note

During 2000–2011, increasing routine measles vaccination coverage worldwide, combined with regular SIAs in countries lacking high coverage with 2 doses of MCV, contributed to a 65% decrease in reported measles incidence and a 71% reduction in estimated measles mortality. Measles elimination has been achieved and maintained in AMR (6), and WPR is approaching its measles elimination goal. However, since 2008, large outbreaks of measles in AFR, EMR, EUR, and SEAR have stalled progress toward regional measles control and elimination targets.

Field investigations of recent measles outbreaks found most cases were among unvaccinated persons, suggesting the main underlying cause was persistent gaps in immunization coverage, despite overall increased measles vaccine coverage (7,8). All five countries with the largest number of infants who did not receive MCV1 through routine immunization services in 2011 had large outbreaks of measles during 2011, highlighting the importance of a strong immunization system. In addition, poor quality SIAs and delays in planned SIAs have resulted in low coverage, contributing to the increased number of measles-susceptible children and ongoing measles virus transmission.

In 2011, estimated global measles mortality increased from the 2010 estimate, and 99% of the measles mortality burden was in AFR, EMR, India, and other SEAR countries. In India, the 36% decrease in estimated measles mortality during 2001–2011 mainly resulted from the National Measles Catch-up Programme to provide MCV2, beginning in 2010, with MCV2 introduction in routine services in states with reported MCV1 coverage ≥80%, and with SIAs followed by MCV2 introduction in routine services in states with reported MCV1 coverage <80%. To prevent measles epidemics and associated morbidity and mortality, WHO recommends that all children receive 2 doses of measles-containing vaccine (9).

What is already known on this topic?

During 2000–2010, global coverage with the first dose of measles-containing vaccine (MCV1) increased from 72% to 85%, >1 billion children received a second opportunity for measles immunization during measles supplemental immunization activities, and global reported measles cases decreased until 2008, then increased in 2010. By 2010, 40% of countries had not met the incidence target of <5 cases per million. As milestones toward eventual global measles eradication, the 2010 World Health Assembly endorsed a series of targets to be met by 2015.

What is added by this report?

The Global Vaccine Action Plan (GVAP) will monitor progress toward achievement of regional measles elimination targets. Estimated global MCV1 coverage increased from 72% in 2000 to 84% in 2011, and the number of countries providing a second dose of measles-containing vaccine (MCV2) through routine services increased from 97 (50%) in 2000 to 141 (73%) in 2011. During 2000–2011, annual reported measles incidence decreased 65%, from 146 to 52 cases per million population, and estimated measles deaths decreased 71%, from 542,000 to 158,000.

What are the implications for public health practice?

During 2010–2011, measles incidence has increased and large measles outbreaks have been reported in multiple countries. To resume progress toward achieving regional measles elimination targets, national governments and partners are urged to ensure that these efforts receive high priority and adequate resources to achieve GVAP targets.

The findings in this report are subject to at least three limitations. First, vaccination coverage estimates in this report include biases resulting from inaccurate estimates of the sizes of the target populations, inaccurate reporting of doses delivered, and inclusion of SIA doses given to children outside the target age group. Second, biases in surveillance data can occur because not all patients seek care and not all of those who seek care are reported. The use of measles surveillance data to estimate measles mortality improved on previously used methods that did not account for the effect of periodic outbreaks on mortality. Finally, the accuracy of the measles mortality model results is affected by biases in all model inputs, including country-specific measles vaccination coverage and measles case-based surveillance data.

In April 2012, the Measles and Rubella Initiative¶¶ launched the 2012–2020 Global Measles and Rubella Strategic Plan to integrate rubella and measles elimination efforts, and provide strategies and guiding principles to resume progress toward regional measles elimination targets (10). The GVAP for the 2011–2020 Decade of Vaccines*** provides strategic objectives and recommended activities for increasing ownership, accountability, and vaccination coverage, as well as indicators for monitoring their impact through achievement of regional measles elimination targets (2). The GAVI Alliance commitment in 2012 to support eligible countries to introduce rubella vaccine using combined measles-rubella SIAs targeting children aged 9 months–14 years provides a unique opportunity to boost population immunity to both measles and rubella.††† The combination of new resources from immunization partners and commitments by countries to fully implement measles control and elimination strategies will help resume progress toward achieving regional measles targets.

## Figures and Tables

**FIGURE:**
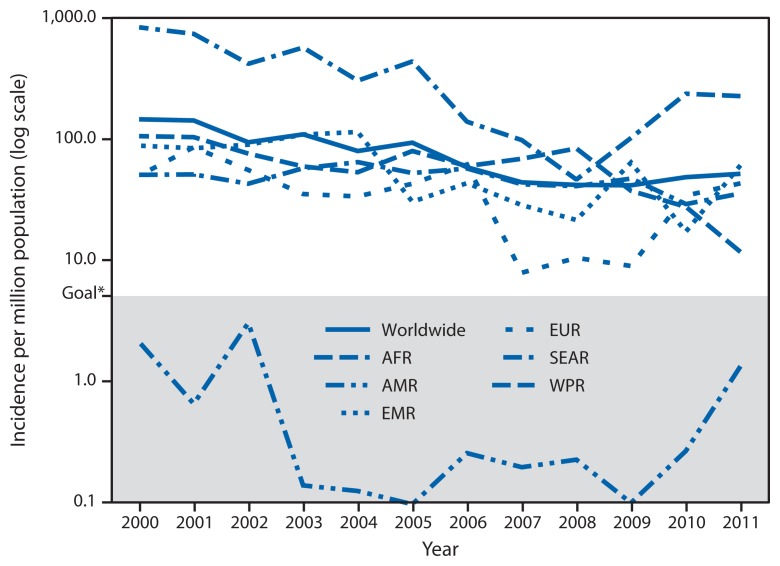
Reported measles incidence per million population, by World Health Organization region and worldwide, 2000–2011 **Abbreviations:** AFR = African; AMR = Americas; EMR = Eastern Mediterranean; EUR = European; SEAR = South-East Asia; WPR = Western Pacific. * As a milestone to measles eradication, the World Health Organization has set a goal of reducing the global incidence of measles to <5 cases per million population by 2015.

**TABLE 1:** Estimates of coverage with the first dose of measles-containing vaccine (MCV1) administered through routine immunization services among children aged 1 year, reported measles cases, and incidence, by World Health Organization (WHO) region, 2000 and 2011

WHO region	2000						2011									
	
	% coverage with MCV1*	No. of reported measles cases†	Measles incidence (cases per million population)§	% countries with incidence <5 per million	Estimated measles deaths		% coverage with MCV1*	No. of reported measles cases†	% decline from 2000	Measles incidence (cases per million population)§	% decline from 2000	% countries with incidence <5 per million	Estimated measles deaths		% mortality reduction 2000 to 2011	% total measles deaths in 2011
	
					No.	(95% CI)							No.	(95% CI)		
African	54	520,102	838	8	338,000	(216,000–736,000)	75	194,364	63	227	73	46	55,000	(23,000–338,000)	84	35
Americas	92	1,755	2.1	89	<100	—	92	1,372	22	1.5	31	94	<100	—	—	0
Eastern Mediterranean	72	38,592	88	17	54,000	(32,000**–**100,000)	83	35,923	7	61	31	45	30,000	(19,000–56,000)	**45**	19
European	91	37,421	50	45	400	(100–2,400)	94	37,073	1	43	14	44	100	(0–180)	62	0
South-East Asia	61	78,558	51	0	137,000	(95,000–205,000)	79	65,161	17	36	30	27	71,000	(52,000–100,000)	52	45
South**-**East Asia (excluding India)	77	39,723	80	0	49,000	(24,000–97,000)	93	35,822	10	61	24	30	15,000	(8,000–30,000)	70	9
India	55	38,835	37	0	88,000	(71,000–108,000)	74	29,339	24	24	36	0	56,000	(44,000–70,000)	36	35
Western Pacific	85	177,052	106	30	13,000	(4,000**–**46,000)	96	21,050	88	12	89	62	1,000	(200**–**30,000)	90	1
**Total**	**72**	**853,480**	**146**	**38**	**542,000**	**(347,000–1,091,000)**	**84**	**354,922**	**58**	**52**	**65**	**55**	**158,000**	**(94,000–527,000)**	**71**	**100**

**Abbreviation:** CI = confidence interval.

* Coverage data: WHO/UNICEF estimates of national immunization coverage. Geneva, Switzerland: World Health Organization; 2012. Available at http://www.who.int/immunization_monitoring/routine/immunization_coverage/en/index4.html.

† Reported case data source: Measles reported cases. Geneva, Switzerland: World Health Organization; 2011. Available at http://apps.who.int/immunization_monitoring/en/globalsummary/timeseries/tsincidencemea.htm. Americas 2011 data source: Measles, rubella, and congenital rubella syndrome surveillance data tables. Washington, DC: Pan American Health Organization; 2012. Available at http://ais.paho.org/phip/viz/im_vaccinepreventablediseases.asp.

§ Population data: United Nations, Department of Economic and Social Affairs, Population Division (2011). World population prospects: the 2010 revision, CD-ROM edition. Any country not reporting data on measles cases for that year was removed from the numerator and denominator.

**TABLE 2:** Measles supplementary immunization activities (SIAs*) and the delivery of other child health interventions, by country — World Health Organization (WHO) regions, 2011

			Children reached in targeted age group		Other interventions delivered					
				
WHO region/ country	Age group targeted	Extent of SIA*	No.	(%)†	Oral polio vaccine	Vitamin A	Insecticide-treated bednets	Deworming medication	Tetanus toxoid vaccination	Rubella vaccination
**Africa**										
Angola	9–59 mos	National	4,635,248	(85)	Yes	Yes		Yes		
Benin	9–59 mos	National	1,411,065	(104)						
Burkina Faso	9–59 mos	National	2,865,517	(113)						
Central African Republic	6–47 mos	National	516,563	(84)	Yes	Yes		Yes		
Côte d’Ivoire	9–59 mos	National	5,820,653	(95)	Yes					
Democratic Republic of the Congo	Varied by province	Rollover — national§	16,793,925	(99)	Yes					
Equatorial Guinea	9–47 mos	Rollover — national	11,658	(50)						
Ethiopia	9–47 mos	Rollover — national and subnational¶	7,806,201	(96)						
Gambia	9–59 mos	National	294,579	(95)		Yes				
Liberia	9–59 mos	National	574,458	(103)	Yes	Yes		Yes		
Mali	9–47 mos	National	4,616,957	(94)						
Mauritania	9–59 mos	National	510,155	(96)						
Mozambique	9–47 mos	National	3,974,977	(104)		Yes		Yes		
Nigeria	6–59 mos	National	28,483,907	(91)	Yes	Yes	Yes	Yes		
Tanzania	6 mos–15 yrs	National	6,686,683	(97)	Yes					
**Americas**										
Bolivia	2–6 yrs	National	869,377	(95)						Yes
Brazil	1–6 yrs	National	16,813,682	(98)						Yes
Chile	1–5 yrs	National	886,802	(75)						Yes
Costa Rica	1–9 yrs	National	620,209	(94)						Yes
Columbia	10–19 yrs	National	7,801,850	(89)						Yes
Ecuador	6 mos–14 yrs	National	4,700,526	(95)						Yes
Mexico	9 mos–59 yrs	National	7,653,521	(99)						Yes
Peru	1–4 yrs	National	2,033,123	(87)						Yes
**Eastern Mediterranean**										
Afghanistan	9–59 mos and 9 mos–10 yrs	Subnational	1,430,510	(95)	Yes				Yes	
Pakistan	6–59 mos	Subnational	9,679,499	(95)	Yes				Yes	
Saudi Arabia	9 mos–18 yrs	National	8,270,316	(97)					Yes	
Somalia	6–59 mos	Subnational	2,080,546	(90)	Yes	Yes	Yes	Yes	Yes	
South Sudan	6–59 mos and 6 mos–14 yrs	National	1,513,864	(97)	Yes					
Sudan	9–59 mos	Rollover — national	5,073,092	(99)	Yes					
Yemen	9–59 mos and 6 mos–14 yrs	Subnational	157,146	(63)	Yes					
**Europe**										
Uzbekistan	1–14 yrs	National	7,502,957	(99)						Yes
**South-East Asia**										
India	9 mos–10 yrs	Rollover — national	30,628,456	(90)						
Indonesia	9–59 mos	Rollover — national	11,544,190	(97)	Yes					
Timor Leste	6 mos–14 yrs	National	454,209	(92)						
**Western Pacific**										
Cambodia	9–59 mos	National	1,504,216	(100)	Yes	Yes		Yes		
Federated States of Micronesia	12–83 mos	Rollover — national	4,889	(96)		Yes		Yes		Yes
Laos	9 mos–19 yrs	National	2,614,002	(97)						Yes
Papua New Guinea	6–35 mos	Rollover — national	464,973	(83)		Yes				
Philippines	9 mos–8 yrs	National	15,649,907	(84)		Yes	Yes	Yes		Yes
**Total**			**224,954,408**							

* SIAs generally are carried out using two approaches. An initial, nationwide catch-up SIA targets all children aged 9 months to 14 years, with the goal of eliminating susceptibility to measles in the general population. Periodic follow-up SIAs then target all children born since the last SIA. Follow-up SIAs generally are conducted nationwide every 2–4 years and generally target children aged 9–59 months; their goal is to eliminate any measles susceptibility that has developed in recent birth cohorts and to protect children who did not respond to the first measles vaccination. The exact age range for follow-up SIAs depends on the age-specifc incidence of measles, first dose of measles-containing vaccine coverage, and the time since the last SIA.

† Values >100% indicate that the intervention reached more persons than the estimated target population.

§ Rollover national campaigns started the previous year or will continue into the next year.

¶ Subnational campaigns were in response to large measles outbreaks (Afghanistan, Ethiopia, Somalia, and Yemen) or natural disasters (Pakistan).

## Footnotes

* Measles elimination is defined as the absence of endemic measles transmission in a defined geographic area (e.g., region or country) for ≥12 months in the presence of a well-performing surveillance system.

† Target dates for measles elimination have been set by four additional WHO regions: 2012 in the Western Pacific Region, 2015 in the Eastern Mediterranean Region and in the European Region, and 2020 in the African Region. The South-East Asia Region has a target to reduce measles mortality by ≥95% from the 2000 estimate by 2015.

§ WHO/UNICEF estimates of national immunization coverage are available at http://www.who.int/immunization_monitoring/routine/immunization_coverage/en/index4.htm.

¶ SIAs generally are carried out using two approaches. An initial, nationwide catch-up SIA targets all children aged 9 months–14 years, with the goal of eliminating susceptibility to measles in the general population. Periodic follow-up SIAs then target all children born since the last SIA. Follow-up SIAs generally are conducted nationwide every 2–4 years and target children aged 9–59 months; their goal is to eliminate any measles susceptibility that has developed in recent birth cohorts and to protect children who did not respond to the first measles vaccination.

** Data for years before 2004 were not available.

†† Countries without case-based measles surveillance include Algeria, Comoros, Guinea-Bissau, India, Mauritius, Monaco, Morocco, Seychelles, San Marino, Somalia, Sao Tome and Principe, and Thailand.

§§ Countries without access to standardized quality-controlled testing by the WHO Measles and Rubella Laboratory Network in 2011 included Cape Verde, Sao Tome and Principe, and Seychelles.

¶¶ The Measles and Rubella Initiative is a partnership established in 2001 as the Measles Initiative, spearheaded by the American Red Cross, CDC, the United Nations Foundation, UNICEF, and WHO. Additional information available at http://www.measlesinitiative.org.

*** The Decade of Vaccines is a collaboration between the World Health Organization, UNICEF, the Bill & Melinda Gates Foundation, the GAVI Alliance, the U.S. National Institute of Allergy and Infectious Diseases, the African Leaders Malaria Alliance, and others to extend, by 2020 and beyond, the full benefit of immunization to all persons worldwide.

††† Additional information available at http://www.gavialliance.org/library/news/gavi-features/2012/partners-launch-plan-to-eliminate-measles-rubella.
