# Errata

**Published:** 2013-02-08

## Vol. 62, No. 2

In the report, “Global Control and Regional Elimination of Measles, 2000–2011,” errors occurred in the text and in Table 1. On page 27, the sixth sentence of the report should read as follows: “During 2000–2011, annual reported measles incidence decreased 65%, from 146 to 52 cases per million population, and estimated measles deaths decreased 71%, from **548,000** to **158,000**.” On page 28, under Mortality Estimates, the last sentence should read as follows: “During 2000–2011, estimated measles deaths decreased 71%, from **548,000** to **158,000**; all regions and India had substantial reductions in estimated measles mortality, ranging from 36% to 90% (Table 1).”

On page 28, the table title should read as follows: “TABLE 1. Estimates of coverage with the first dose of measles-containing vaccine (MCV1) administered through routine immunization services among children aged 1 year, reported measles cases and incidence**, and estimated measles mortality,** by World Health Organization region, 2000 and 2011.”

In Table 1, in the Eastern Mediterranean row, the number of estimated measles deaths in 2000 should read, “**60,000 (32,000–100,000)**”; the percentage mortality reduction from 2000 to 2011 should read, “**49**.” In the South-East Asia row, the percentage mortality reduction from 2000 to 2011 should read, “**48**.” In the Western Pacific row, the number of estimated measles deaths in 2000 should read, “**13,000 (4,000–65,000)**,” and the number of estimated measles deaths in 2011 should read, “**1,000 (180–44,000)**.” In the Total row, the number of estimated measles deaths in 2000 should read, “**548,000 (347,000–1,109,000)**,” and the number of estimated measles deaths in 2011 should read, “**158,000 (94,000–540,000)**.”

